# Thick primary melanoma has a heterogeneous tumor biology: an institutional series

**DOI:** 10.1186/1477-7819-9-40

**Published:** 2011-04-14

**Authors:** Ari-Nareg Meguerditchian, Kobby Asubonteng, Calvin Young, Bethany Lema, Gregory Wilding, John M Kane III

**Affiliations:** 1 Department of Surgery, McGill University, Montreal, QC H3A 1A1, Canada; 2 Department of Biostatistics, State University of New York, Buffalo, NY, 14214, USA; 3 School of Medicine and Biomedical Sciences, State University of New York, Buffalo, NY, 14214, USA; 4 Department of Surgical Oncology, Roswell Park Cancer Institute, Buffalo, NY 14263, USA

## Abstract

**Background:**

Thick melanomas (TM) ≥4 mm have a high risk for nodal and distant metastases. Optimal surgical management, prognostic significance of sentinel node biopsy (SLNB), and benefits of interferon (IFN) for these patients are unclear. As a continuum of increasing tumor thickness is placed into a single TM group, differences in biologic and clinical behavior may be lost. The purpose of this study was to better characterize the diverse biology in TM, including the value of increasing thickness and nodal status information, potentially identifying high risk TM subgroups that may warrant more aggressive treatment/follow up.

**Methods:**

155 consecutive TM patients treated at a single institution between 1971 and 2007 were retrospectively reviewed. Patient, disease and treatment features were analyzed with respect to disease-free (DFS) and overall survival (OS).

**Results:**

Median patient age was 66 years and 68% of patients were men. The trunk was the most common TM location (35%), followed by the head and neck (29%) and lower extremities (20%). Median thickness was 6 mm and 61% were ulcerated. 6% patients had stage IV disease, 12% had clinical nodal metastases. Clinically negative lymph node basins were treated by observation (22 patients - 15.4%), elective lymph node dissection (ELND) (24 patients - 17.6%) or SLNB (91 patients - 67%). 75% of ELND's and 53% of SLNB's were positive. Completion node dissection was performed in 38 SLNB+ patients and 22% had additional positive nodes. 17% of the study patients received IFN. At median follow up of 26 months, 5 year DFS and OS were 42% and 43.6%. For SLNB positive vs negative, median DFS were 22 vs 111 months (p = 0.006) and median OS were 41 vs 111 months (p = 0.006). When stratified by tumor thickness ≤ vs > 6 mm, 5 year DFS was 58.3% vs 20% (p < 0.0001) and OS was 62% vs 20% (P < 0.0001). IFN had no impact on DFS or OS (p = 0.98 and 0.8 respectively).

**Conclusion:**

Within the high risk group of patients with TM, cases with tumor thickness > 6 mm or a positive SLNB had a significantly worse DFS and OS (p < .0001, <.0001 and .006, .006).

## Introduction

Approximately 5% of newly diagnosed melanomas will have a Breslow thickness ≥ 4 mm (thick melanomas, TM) [1]. As the risk of nodal metastases correlates with primary tumor thickness, 40% of TM patients will develop nodal disease [2]. Increasing tumor thickness is also associated with a higher rate of distant metastases and worse survival, with reported 5 and 10 year overall survival (OS) for TM of 45-79% and 32-44%, respectively [3]. Historically, the role of elective lymph node dissection (ELND) in the management of TM patients has been controversial [4-9]. In addition, very few studies have addressed the prognostic value of sentinel lymph node biopsy (SLNB) in such a high-risk cohort of patients [10-16]. Given the high risk for systemic metastases, the role of SLNB in TM remains controversial. Despite the poor overall outcome for this group, it is still not clear if the benefits of adjuvant interferon therapy outweigh the potential side effects [17-21]. In fact, the assumption that patients with TM have a dismal prognosis has led some to consider any treatment other than local control to be primarily palliative [2].

The purpose of the American Joint Committee on Cancer (AJCC) melanoma staging system is to provide prognostic information for somewhat homogeneously grouped subsets of patients. In an effort to maintain simplicity, the continuous variable of tumor thickness has been converted into categorical variables (< 1 mm, 1.01-2 mm, 2.01-4 mm, > 4 mm) [22]. As a limitation of this methodology, it is hypothesized that thick primary tumors (≥ 4 mm) may actually represent a larger and more diverse group of biologic behaviors as compared to other primary thickness categories given that the thickness range (4 to infinity) is much greater than the 1-2 mm intervals in the other groups. Therefore, the current study examines whether TM has a uniformly poor prognosis versus a heterogeneous tumor biology and if nodal status has additional prognostic value in this high risk subset of patients.

## Methods

After Institutional Review Board approval, cases of primary cutaneous melanoma ≥ 4 mm (TM) treated at Roswell Park Cancer Institute between 1971 and 2007 were identified through the Tumor Registry. Patient demographic data, clinical tumor features, surgical treatment, and the final pathology were retrospectively obtained from the medical record. Information regarding adjuvant therapy, locoregional recurrence, distant relapse, and survival was extracted from the outpatient follow-up records. Laboratory tests and imaging were used selectively, based upon clinical examination and symptoms. Patients underwent wide excision and those with clinically positive lymph nodes were treated by therapeutic lymph node dissection. Before 1996, patients with clinically negative lymph node basins were treated by observation or ELND. After that date, SLNB was the standard of care at Roswell Park Cancer Institute. Nodal dissections were performed according to well-described surgical techniques [23]. Lymph node analysis was done by bi-valving nodes identified in the dissection specimen, whereas sentinel lymph nodes were analyzed by serial sectioning. Our institution's approach has been to present these cases at multidisciplinary tumor conferences. Generally, radiation therapy is offered to patients with bulky nodes (≥3 cm), nodes showing evidence of extra-capsular invasion or multinodal disease (≥ 4 nodes). Other than clinical trials, adjuvant interferon is presented as an option to these patients with a frank discussion on potential side effects and ambiguity on certain aspects of treatment benefits. Longitudinal follow up was performed based upon National Comprehensive Cancer Network guidelines [24], with every 3-4 month follow-up until year two, followed by every 6 month visits until year five and annual visits thereafter. Most patients received their long-term cancer follow-up at Roswell Park Cancer Institute. For those that did not, the institutional cancer registry performed annual mailings to update the records of these patients.

Primary tumor site was defined as: 1) head and neck (including scalp and cervical areas); 2) trunk (anterior, posterior and lateral surfaces of chest and abdomen from the clavicles to the inguinal ligaments); 3) upper extremity (including the shoulder); and 4) lower extremity (below the inguinal ligament). All patients were staged using the AJCC 7th edition based upon primary tumor features, clinical or pathologic regional lymph node status (where available), and the presence or absence of distant metastases at the time of initial diagnosis. Disease-free survival (DFS) and overall survival (OS) were calculated from the time of diagnosis until relapse or death, respectively.

Statistical analysis was performed using SAS 9.1 software (SAS Institute, Cary, NC, USA). Descriptive statistics were used to report patients' baseline characteristics. Estimation of the overall and disease free survival distributions was done using the Kaplan-Meier method. Assessment of observed overall group differences in the survival distributions was done using the log-rank test. Follow-up testing between pairs of groups was done in conjunction with a Bonferroni correction. Multivariate analyses were done using the Cox proportional hazard model. A 0.05 nominal significance level was used.

## Results

### Patient and tumor characteristics

One hundred fifty-five patients were identified with TM; 106 males (68%) and 49 females (32%). Median age was 66 years (range 18 - 95 years). The most common primary tumor site was the trunk (35%) followed by head and neck (29%), lower extremity (20%), and upper extremity (16%). Median tumor thickness was 6.0 mm (range 4.0 - 50.0 mm), mean was 7.8 mm, and 18% of patients had a primary lesion ≥ 10 mm. Ulceration was present in 61% of cases and 87% of tumors were Clark level IV or V. The AJCC stage at presentation is shown in table 1. Synchronous distant metastases were present in 6% of TM patients (n = 9). In total, 146 patients with stage III or lesser TM were offered definitive surgical therapy.

**Table 1:** AJCC stage (7^th ^edition) at presentation (and following lymph node staging) for patients with thick melanoma (≥ 4 mm)

AJCC STAGE	N (%)
IIB	35 (22.8%)

IIC	46 (29.5%)

IIIA	21 (13.4%)

IIIB	25 (16.1%)

IIIC	19 (12.1%)

IV	9 (6%)

### Primary surgical and adjuvant therapy

All patients without distant metastases underwent wide excision of the primary tumor site as the potentially curative surgical therapy. Nodal metastatic disease was clinically obvious at presentation in 12% of patients (n = 9). These patients underwent therapeutic lymph node dissection. The remaining 137 patients were treated with nodal basin observation (15.4%), ELND (17.6%) or SLNB (67%) after 1996. Five patients treated by nodal basin observation were subsequently diagnosed with nodal disease, a mean of 20.4 months after having undergone surgery (range: 4-38). Nodal metastases were found in 75% (18/24) of the patients undergoing ELND. Thirty-three percent had only one positive lymph node; the remainder had 2-6 positive nodes. Fifty-three percent (48/92) of the SLNB patients had a positive sentinel lymph node. The majority of patients had only one positive sentinel node, however, 27.5% had ≥ 2 positive sentinel lymph nodes. A completion lymph node dissection (CLND) was performed in 38 of the positive sentinel lymph node biopsy patients and 22% had additional positive nodes (range 1-28) on final pathology. If the results of ELND and SLNB are combined, 60% (69/115) of patients with a clinically negative nodal basin had pathologically confirmed nodal metastatic disease (median number of positive nodes 1, range 1-28). Due to a concern for locoregional recurrence, 20% of patients (n = 29) received adjuvant radiation therapy. Adjuvant interferon therapy was administered to 17% of patients (n = 24).

### Outcome and survival

**Figure 1:**
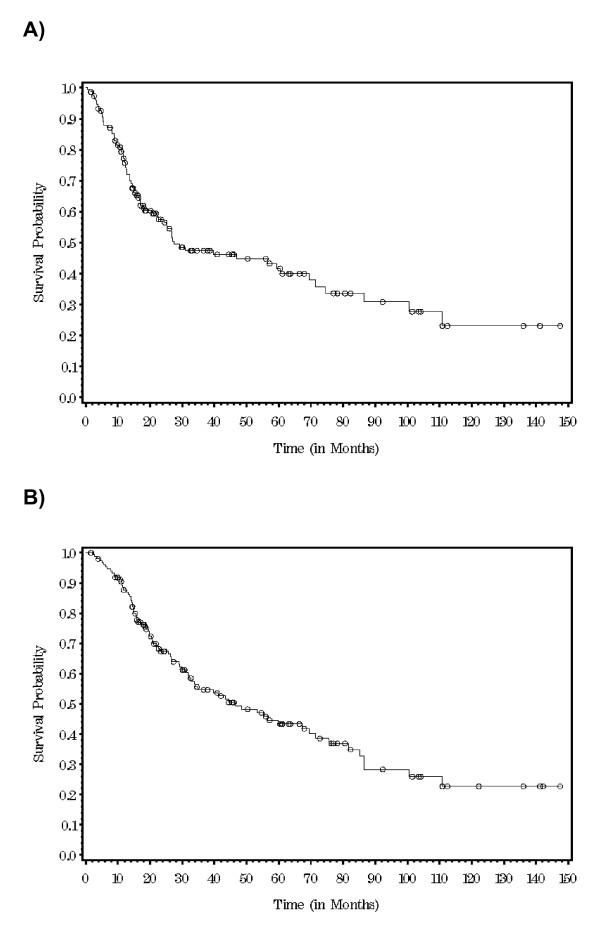
**Kaplan-Meier survival curves for disease-free survival (A) and overall survival (B) in patients with thick melanoma (≥ 4 mm) **.

Median follow-up was 26 months (range 1-148); 23% of the study population had a follow-up ≥ 5 years. For the non-stage IV patients, 47.3% (69/146) developed a recurrence. The most common sites of first recurrence were 28.7% primary tumor site/in transit disease, 19.7% regional lymph node basin, 17.4% pulmonary, 17.4% brain, 10.1% peritoneal surface/abdominal viscera, and 8.7% bone. DFS at 5 and 10 years were 42% and 23.5%, respectively. OS at 5 and 10 years were 43.6% and 24%, respectively. Kaplan-Meier survival curves for DFS and OS are shown in figure 1.

**Figure 2:**
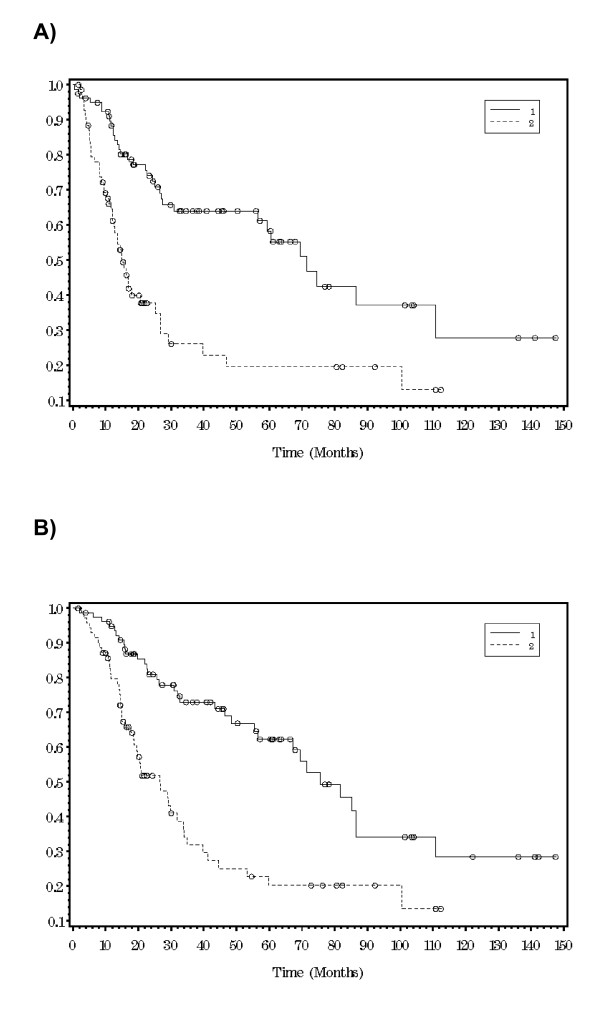
**Kaplan-Meier survival curves for disease-free survival (A) and overall survival (B) in patients with thick melanoma stratified by primary tumor thickness ≤ 6 mm (group 1) versus > 6 mm (group 2) **.

Differences in DFS and OS were observed based upon the primary tumor site with the lower extremity having the most favorable outcome at 5 years (DFS 55%, OS 62%) and the trunk having the worst (DFS 33%, OS 33%). However, these differences did not reach statistical significance (p = 0.07 for both DFS and OS). As expected, stage IV patients did the worse, with a mean survival less than 21 months. However, even within non-metastatic patients, AJCC stage was a statistically significant predictor of DFS and OS (table 2). Given that the median primary tumor thickness was 6 mm, DFS and OS were reanalyzed based upon two subgroups: ≤ 6 mm (54%) versus > 6 mm (46%). Kaplan-Meier survival curves for DFS and OS based on these subgroups are shown in figure 2. Both DFS and OS were statistically significantly higher in patients with a melanoma ≤ 6 mm versus > 6 mm (table 3). An additional observation is that the rates of change in DFS and OS over time were not the same for both groups, as reflected by 1, 5 and 10 year survival. For TM ≤ 6 mm, over half of patients were still alive and disease-free at 5 years, but this number decreased by an additional 50% over the next 5 years. In contrast, for TM > 6 mm, 80% of patients died by 5 years with very few additional recurrences or deaths after that time. In terms of adjuvant therapy, interferon had no significant impact on DFS and OS (p = 0.98 and 0.8, respectively).

**Table 2:** Disease-free and overall survival by AJCC stage (7^th ^edition) for non-metastatic patients with thick melanoma (≥ 4 mm)

AJCC STAGE	DISEASE-FREE SURVIVAL		OVERALL SURVIVAL	
	
	5 year	10 year	5 year	10 year
**IIB**	56.9%	42.3%	58.2%	43.1%

**IIC**	42.8%	0%	45.5%	12.1%

**IIIA**	40.8%	18.2%	54.6%	20.3%

**IIIB**	30.3%	30.3%	33.0%	33.0%

**IIIC**	10.0%	0%	13.1%	0%

***p value***	0.0001		0.0004	

**Table 3:** Disease-free and overall survival in patients with thick melanoma stratified by primary tumor thickness ≤ 6 mm versus > 6 mm

	DISEASE FREE SURVIVAL		
	
	1 year	5 year	10 year
≤ **6 mm **(54%)	88.4%	58.4%	27.9%
**> 6 mm **(46%)	62.9%	19.6%	13.0%

*p < 0.0001*			

	**OVERALL SURVIVAL**		
	
	**1 year**	**5 year**	**10 year**

≤ **6 mm **(54%)	94.9%	62.4%	28.4%

**> 6 mm **(46%)	79.6%	20.3%	13.5%

*p < 0.0001*			

**Figure 3:**
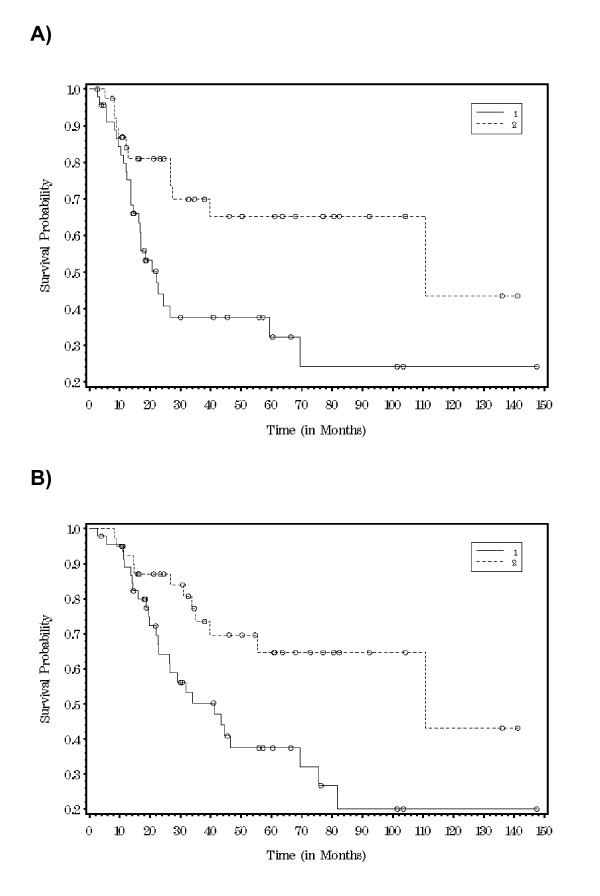
**Kaplan-Meier survival curves for disease-free survival (A) and overall survival (B) in patients with thick melanoma stratified by a positive (group 1) versus negative (group 2) sentinel lymph node biopsy **.

DFS and OS were also analyzed based upon a positive versus negative SLNB in the 92 patients who underwent this procedure. The presence of a positive SLNB was significantly associated with an approximately 50% reduction in DFS and OS at both 5 and 10 years (p values 0.006 and 0.006, respectively). Survival rates according to SLNB status as well as the Kaplan-Meier survival curves are shown in table 4 and figure 3.

**Table 4:** Disease-free and overall survival in patients with thick melanoma stratified by a positive versus negative sentinel lymph node biopsy (n = 92)

	DISEASE FREE SURVIVAL		OVERALL SURVIVAL	
	
	5 year	10 year	5 year	10 year
**SLNB +**	32%	20%	37%	24%

**SLNB -**	65%	43%	65%	44%

	*p = 0.006*		*p = 0.006*	

## Discussion

Many aspects of the optimal curative surgical management of melanoma ≥ 4 mm remain to be clarified [2,8,10,25]. Specifically, only a few publications have addressed the value of SLNB in this high risk population, despite the fact that it is a well-established component of the contemporary management of intermediate thickness melanoma [10-14].

The results of our study challenge the notion that all patients with TM have uniformly aggressive disease. Instead, given that this broad group encompasses melanoma thickness from 4 mm to infinity (a continuous variable converted into a categorical variable), one would expect a heterogeneous tumor biology with differing prognoses. For the entire group, DFS and OS at all time points were almost identical. Therefore, clinical recurrence is usually synonymous with death in this cohort of patients. However, 5-year OS for patients presenting without metastases was 46-57% and was still a reasonable 13-55% even in the presence of nodal/lymphatic metastases. These outcomes are equivalent or superior to the 5 year survival rates for primary pancreas or esophageal cancer (which are treated very aggressively from a surgical standpoint). Furthermore, when outcome is analyzed based upon tumor thickness subsets of ≤ 6 mm versus > 6 mm, differences in biologic behavior and survival become readily apparent. For patients with melanoma ≤ 6 mm, the risk of dying was much lower at 5 years, but did not significantly decrease over time. In contrast, the vast majority of patients with melanoma > 6 mm died within the first 5 years with very few deaths after that point. Given the very early aggressive behavior of "ultra-thick" melanomas (> 6 mm), this patient population may be the ideal group to study potential adjuvant systemic therapies as there will be a significant number of events in a short period of time. In terms of cancer surveillance, it would be important to follow the ≤ 6 mm TM patients long-term as they have a risk for recurrence and death past 5 years that is similar to patients with intermediate thickness melanoma.

The presence and extent of lymph node metastases is clearly associated with decreased survival in melanoma [26]. In the absence of clinically apparent nodal metastatic disease, SLNB has become the standard approach for regional lymph node staging. In fact, a positive sentinel lymph node biopsy is one of the most powerful predictors of outcome in melanoma patients [27-29]. Unfortunately, many of the large multi-center SLNB trials focused mainly on patients with intermediate thickness tumors (1.01-<4 mm). To date, there have been few studies that examined the role of SLNB in the management of TM; 131 patients in Gershenwald et al., 126 patients in Ferrone et al., and 114 patients in Carlson et al [12-14]. These studies were strikingly similar in terms of patient and tumor characteristics, rates of SLNB positivity, and survival. In addition, SLNB status and ulceration were statistically significant independent predictors of survival with SLNB status being the most powerful variable. Although thickness stratification was not examined in Gershenwald et al. and was not associated with overall survival in Carlson et al. (82% for ≤6 mm versus 61% for >6 mm, p = 0.085), Ferrone et al. found that stratified tumor thickness (≤5.5 mm versus >5.5 mm) and age (<60 versus ≥60) were also independent prognostic variables and could be combined with SLNB status and ulceration to create a prognostic model. The conclusion in these 3 studies was that SLNB does have prognostic value in the staging of patients with TM. In support of the findings from the above studies, we also found that a positive SLNB was associated with a significantly increased risk for recurrence and death (table 4 and figure 2).

A noteworthy observation from our study is that 72.5% of our positive SLNB patients ultimately had only one positive node and only 22% of the CLND specimens had additional positive nodes on final pathology. Interestingly, these findings are almost identical to the 70.5% single positive SLNB rate reported in the Multicenter Selective Lymphadenectomy Trial-I (MSLT-I) for lower risk patients with intermediate thickness melanoma (1.2-3.5 mm)[28]. The rate of additional positive nodes at CLND for a positive SLNB for Ferrone et al. was 21% and a similar 16% positive CLND rate was seen in the Sunbelt Melanoma Trial for a much more diverse group of melanoma patients (tumors ≥1 mm)[14,30]. In addition, recent data for a subset of TM patients in the Sunbelt Melanoma Trial also reported no additional positive nodes in 82% of CLND [31]. Therefore, despite the fact that TM has been historically associated with an anticipated high risk for nodal metastatic disease, it is almost certainly a more diverse group of tumor biologies with subsets that may behave akin to lower risk, intermediate thickness melanomas. Consequently, clinical trials examining the treatment of nodal metastatic disease and the efficacy of adjuvant therapies should not exclude TM patients simply based upon tumor thickness alone.

Although the results and conclusions of our current study are similar to some of the TM SLNB studies above, there are several key differences. First, our patient population contained all patients diagnosed with TM, including synchronous clinical nodal and distant metastatic disease. This allows for a broader understanding of the true "natural history" of patients presenting with TM. Second, our rate of SLNB positivity was higher than in the other studies (55% versus 30-39%). However, this would be anticipated given the fact that we had a higher risk TM patient population in terms of median tumor thickness (6 mm versus 5-5.5 mm) as well as ulceration (61% versus 35.1-50%). Third, as opposed to looking at SLNB status and ulceration as individual variables, we incorporated those factors into the patients' AJCC stage as a way to provide more accurate, clinically relevant survival information for the clinician treating a newly diagnosed TM patient. Finally, similar to only Ferrone et al., we found that stratification by tumor thickness (≤6 mm versus >6 mm) was significantly associated with both DFS and OS. In fact, it identified what appears to be two very different tumor biologies as noted above; an aggressive, "ultra-thick" group that will succumb to distant metastatic disease within a few years of diagnosis and a second, somewhat more indolent group that has a reasonable long-term prognosis but a prolonged risk for recurrence and death that is similar to other melanoma thickness subsets.

Controversy surrounding the benefit of adjuvant interferon in "high risk" melanoma patients continues [7,17,18]. In our study, only 17% of patients received interferon and this was not associated with a statistically significant improvement in survival. Lack of a statistically significant impact on recurrence and death from melanoma in a similar group of patients has also been reported by Gajdos et al [32]. Although outside the context of a clinical trial, the survival results reported here demonstrate that, even within the same high risk group of patients as defined by tumor thickness ≥4 mm (which was a cohort in the Eastern Cooperative Oncology Group EST-1684 study), there was tremendous variability in prognosis especially with long-term follow-up. A prolonged risk of recurrence past 5 years in subsets of TM patients should be considered when discussing the potential overall survival benefits of adjuvant interferon.

## Conclusion

Significant biologic heterogeneity exists within TM patients currently defined simply by tumor thickness ≥4 mm. This translates in differences in clinical outcome both in terms of DFS and OS associated with a threshold thickness of 6 mm. Patients with "ultra-thick" melanoma (>6 mm) are at significant risk for early recurrence and death but may enjoy long-term survival if they make it to 5 years. In contrast, not all patients with a melanoma 4-6 mm thick have limited survival and dismal long-term prognosis. However, their risk of recurrence continues past 5 years. SLNB status is a powerful prognostic variable in TM. Consequently, the surgical treatment of newly diagnosed TM patients without clinical distant metastatic disease should be identical to that of intermediate thickness melanoma patients; wide excision, SLNB, and formal lymphadenectomy for either a positive SLNB or clinically apparent nodal metastatic disease. One should strongly consider the continuum of thickness as well as nodal status when assessing TM patients for adjuvant therapy or designing clinical trials for a "high risk" melanoma population.

## Abbreviations

TM: Thick melanoma, AJCC: American Joint Committee on Cancer, SLNB: sentinel lymph node biopsy, CLND: Completion lymph node dissection, ELND: Elective lymph node dissection, DFS: Disease-free survival, OS: Overall survival.

## Competing interests

The authors declare that they have no competing interests.

## Authors' contributions

ANM participated in study conception and design, data acquisition, analysis and interpretation, manuscript drafting and final approval; KA participated in data analysis and interpretation, manuscript drafting and final approval CY participated in data acquisition and analysis, manuscript drafting and final approval; BL participated in data acquisition and analysis, manuscript drafting and final approval; GW participated in study design, data analysis and interpretation, manuscript drafting and final approval; JMK participated in study conception and design, data acquisition, analysis and interpretation, manuscript drafting and final approval
